# Antifungal and antiproliferative activities of endophytic fungi isolated from the leaves of *Markhamia tomentosa*

**DOI:** 10.1080/13880209.2016.1263671

**Published:** 2016-12-09

**Authors:** Mutiat Ibrahim, Nutan Kaushik, Abimbola Sowemimo, Hemraj Chhipa, Trevor Koekemoer, Maryna van de Venter, Olukemi A. Odukoya

**Affiliations:** a Department of Pharmacognosy, Faculty of Pharmacy, University of Lagos, College of Medicine campus, Lagos, Nigeria;; b The Energy and Resources Institute (TERI), Indian Habitat Centre, New Delhi, India;; c Department of Biochemistry and Microbiology, Nelson Mandela Metropolitan University, Port Elizabeth, South Africa

**Keywords:** *Trichoderma longibrachiatum*, *Syncephalastrum racemosum*, pathogenic fungi, HeLa cancer cell line, *Spodoptera litura* larvae

## Abstract

**Context:** Plants harbor endophytes with potential bioactivity. *Markhamia tomentosa* (Benth) K. Schum ex. Engl. (Bignoniaceae) is reported to possess antioxidant, anti-inflammatory and anticancer activities.

**Objective:** The antifungal and antiproliferative properties of endophytic fungi extracts and fractions from *M. tomentosa* were evaluated.

**Material and methods:** Endophytic fungi were isolated from the leaves of *M. tomentosa* and identified by ITS-rDNA sequence analysis. The antagonistic effect of the fungal strains was investigated against pathogenic fungi viz, *Fusarium oxysporum, Sclerotinia sclerotiorium, Rhizoctonia solani,* and *Botrytis cinerea* using the dual culture assay for 5–7 days. Antiproliferative effect of the fungal extracts and fractions (3.91–250 μg/mL) on HeLa cancer cell line was tested and IC_50_ was calculated. Poisoning food assay and antifeedant activity against the pathogenic fungi and *Spodoptera litura* larvae, for 7 days and 2 h, respectively, was also tested at concentrations of 250, 500 and 1000 μg/mL.

**Results:** Fungal endophytes *Trichoderma longibrachiatum* and *Syncephalastrum racemosum* were isolated from the leaves of *M. tomentosa*. Isolated endophytic fungal strains and solvent extracts showed MIC value of 1000 μg/mL against tested pathogenic fungi in the dual culture and poisoning food assays. Methanol fraction of *S. racemosum* isolate showed the most effective antiproliferative activity with IC_50_ of 43.56 μg/mL. Minimal feeding deterrent activity against *S. litura* larvae was also observed.

**Discussion and conclusion:** These findings showed that the leaves of *Markhamia tomentosa* harbor strains of endophytic fungi with promising health benefits, and suggest their antifungal and antiproliferative effects against pathogenic fungi and HeLa cancer cell line.

## Introduction

Endophytes are extremely common and highly diverse microorganisms that live within healthy tissues of the host without causing visible symptoms of plant diseases (Borges et al. 2009). Both fungi and bacteria are the most common microbes existing as endophytes, but the most frequently isolated are the endophytic fungi (Staniek et al. 2008). Traditionally, endophytic fungi have been considered as plant mutualists, benign commensals, or latent pathogens, and are currently viewed as an outstanding source of bioactive natural products (Vega et al. 2008). It is reported that medicinal plants with an ethnobotanical history are known to harbour endophytic fungi which are considered as rich source of novel bioactive products with antimicrobial, insecticidal, and anticancer activities (Strobel & Daisy 2003; Yu et al. 2010; Kharwar et al. 2011; Han et al. 2013). Some fungal endophytes have been reported to play a significant role in plant resistance to insects by altering the nutritional quality of plants or by production of various alkaloid-based defensive compounds, e.g., the endophytic fungus, *Phomopsis oblonga* reported to be responsible for the reduction in the spread of Dutch elm disease causing agent *Ceratocystis ulm* by controlling its vector, *Physocnemum brevilineum* and Muscodor vitigenus from the liana plant *Paullina paullinioides* active against common mothballs (Webber 1981; Daisy et al. 2002; Dutta et al. 2014). The intrinsic nature of the interactions among endophytes, host plants, and pests which are mediated by the bioactive compounds is an area of future discoveries (Nicoletti & Fiorentino 2015). This had led to the focus of many scientists on fungal endophytes as potential source of novel and biologically active compounds that demonstrate antimicrobial, anti-arthritis, immunosuppressive activity, antioxidant, insecticidal as well as anticancer activities (Carroll 1995; Sadrati et al. 2013).

*Markhamia tomentosa* (Benth) K. Schum. ex. Engl. (Bignoniaceae), a tree found mostly in West Tropical Africa, has been used by local herbalists to treat general body pain, edema, pulmonary troubles, scrotal elephantiasis and cancer (Burkill 1985). The antioxidant, antimicrobial, antiulcer, anti-inflammatory, as well as the analgesic activities of the ethanolic extract of *M. tomentosa* have been reported (Aladesanmi et al. 2007; Tantangmo et al. 2010; Temdie et al. 2012; Sowemimo et al. 2013; Sofidiya et al. 2014).

In our earlier work, we reported the antiproliferative and apoptosis inducing activity of *M. tomentosa* leaf extract against HeLa cancer cell lines (Ibrahim et al. 2013). Thus, in this present study, we aim to isolate and identify the endophytes residing in the leaves of the medicinal plant, *M. tomentosa* and to investigate the antifungal, antiproliferative, and antifeedant activities of the endophytic fungal extracts and fractions.

## Materials and methods

### Collection of plant sample

Fresh leaves of *M. tomentosa* were collected from Oke-igbo, Ondo state, Nigeria in July, 2013. The taxonomic identification and authentication of the plant was carried out by Mr. Oyetola Oyebanji at the Herbarium of the Department of Botany and Microbiology, University of Lagos where a voucher specimen (LUH 5535) was deposited. The plant sample was kept in a sealed plastic bag and returned to the laboratory on the same day for the isolation of the endophytic fungi.

### Surface sterilization and isolation of pure fungal strain

Within 8 h of collection, the plant samples were washed under running tap water to remove dust and debris in the laboratory. The plant samples were sterilized according to the protocol reported by Kjer et al. (2009) and Scalvenzi (2012) with some modifications. Leaf samples were cut into small segments of approximately 1 cm ×1 cm using a sterile razor blade. Under aseptic conditions, the leaf segments were surface-sterilized through sequential immersion in 70% ethanol for 60 s, 10% sodium hypochlorite for 5 min, 70% ethanol for 30 s, followed by a final rinse in sterilized distilled water for 5 min. The samples were air-dried in the laminar flow hood and placed on Petri dishes containing potato dextrose agar and malt extract agar media (previously autoclaved and with addition of 0.01% streptomycin antibiotic) for direct contact of the cut edges with the agar surface. After 3–4 days of incubation at room temperature, the first fungal hyphae were visible from the edge of the samples. Different fungal strains developed from each sample and individual strains were isolated by transferring hyphal tips onto potato dextrose agar and malt extract agar media. This step was repeated several times until pure endophytic fungal strain with uniform colony was achieved.

For tentative identification, microscopic slides for each fungal mycelium were prepared by staining with lactophenol cotton-blue. The slides were mounted in polyvinyl lactic acid glycerol (PVLG) by heating at 65 °C for 3 days and examined under a light microscope (Kumar & Kaushik 2013).

### Long-term storage of endophytic fungi colony

Pure cultures of each fungal isolate were preserved for long-term use. This was done by transferring small pieces of media supporting pure fungal growth into sterile Eppendorff tubes containing 1 mL of 30% (v/v) sterilized glycerol solution and sterilized rice media. Growth of fungal strains in both media was observed after approximately 4 days. Glycerol stock solution and rice culture media were maintained and transferred to the deep freezer at −20 °C and refrigerator at 4 °C, respectively for future use.

### Molecular identification of endophytic fungi strains

#### Reagents and solutions

Extraction buffer [200 mM Tris-HCl (pH 7.5), 25 mM EDTA, 250 mM NaCl, 0.5% SDS]; cold phenol:chloroform (1:1); chloroform; cold iso-propanol; cold 70% ethanol; 10 × PCR buffer [1 × contains 10 mmol/L Tris-HCl, (pH 8.8), 50 mmol/L KCl, 1.5 mmol/L MgCl_2_]; primers ITS 1 (5′-TCCGTAGGTGAACCTGCGG-3′) and ITS 4 (5′-TCCTCCGCTTAT TGATATG-3′); dNTP mix; Tag polymerase; nuclease-free water; 1-1.5% agarose gel; TBE buffer [10 × contains 324 g Tris base, 165 g boric acid, 120 mL 0.5 M EDTA (pH 8.0)].

#### Fungal materials

Purified endophytic fungal strains from the leaves of *M. tomentosa* were grown in potato dextrose broth (PDB) at room temperature for 5 to 7 days. Following the formation of fungal colony, each fungal mycelium was allowed to drain for few minutes and 30–50 mg was harvested into 1.5 mL Eppendorff tube containing 0.5 mL sterile water. The tube was swirled for 30 seconds and centrifuged at 2000 rpm for 5 min. The supernatant was discarded and the pellet (mycelium) was incubated at −80 °C for 24 h. The frozen mycelium was lyophilized and ground with iron beads in tissue-lyser to fine powder (Prabha et al. 2013).

#### Endophytic fungi DNA extraction

The DNA extraction procedure was adopted with some modifications from the standard phenol-chloroform method (Davies et al., 1986; Prabha et al. 2013). The powdered mycelium transferred into 2 mL centrifuge tube was mixed with 500 μL of extraction buffer, vortexed for 5 s and incubated at room temperature for 30 min. The reaction mixture was centrifuged at 13,000 rpm for 1 min. The supernatant was transferred into a new centrifuge tube and equal volume of cold phenol: chloroform was added to it. The mixture was vortexed briefly and centrifuged again at 13,000 rpm for 2 min. The supernatant was transferred into another tube and re-extracted with 300 μL of chloroform. The mixture was vortexed and centrifuged again as done in the previous step. The final supernatant was then transferred into new centrifuge tube and 300 μL of cold iso-propanol was added to it. The mixture was swirled gently and incubated at −80 °C for 30 min. After the incubation period, the mixture was centrifuged at 13,000 rpm for 5 min to recover the nucleic acids as the supernatant was discarded. Finally, the harvested pellet was washed with 70% cold ethanol, air-dried and re-suspended in 100 μL of sterile water. The DNA yield and quality were assessed by standard electrophoresis through a 1% (w/v) ethidium bromide-stained agarose gel.

#### Polymerase chain reaction (PCR)

Specific DNA of each fungus was amplified by PCR in a total volume of 25 μL containing 2.5 μL of 10 × PCR buffer, 0.5 μL of 1 mmol dNTPs, 2.5 μL of 10 pmol ITS1 and ITS 4 primers, 1 μL of 40 ng template DNA and 0.25 μL of 5 units Taq polymerase. PCR amplification was performed in a Veriti thermal cycler (Applied Biosystem, USA) using the following protocols: denaturation at 94 °C for 2 min; 35 cycles of 94 °C for 1 min, primer-specific annealing temperature at 57 °C for 1.30 min and extension at 72 °C for 2 min; a final extension at 72 °C for 4 min. Amplified DNA was resolved by electrophoresis at 80 V for 90 min in a 1.5% agarose gel in 1.0 × TBE buffer. The gel was pre-stained with ethidium bromide and the band to PCR product was photographed under UV transilluminator (UVtec Cambridge, United Kingdom). 

The amplified products were sequenced by Merck sequencing services, Bangalore, India using Sanger method. Endophytic fungi identification was carried out on the basis of similarity of amplified sequence with US National Centre for Biotechnology Information (NCBI) database using Basic Local Alignment Search Tool (nBLAST). Accession numbers of the respective fungal strains are as shown in Table 1.

**Table 1.** Identification and antagonistic activity of endophytic fungi isolated from the leaves of *Markhamia tomentosa*.

				Activity against pathogenic fungi			
Isolate code	Similarity with	% similarity	Accession no	F	R	S	B
MF1	*Trichoderma longibrachiatum* strain BHU-BOT-RYRL17	99	KR856223.1	+	+	+	−
MF3	*Syncephalastrum racemosum* strain AQGSS 12	98	KP721597.1	+	−	−	−
MF5	*Trichoderma longibrachiatum* voucher 50	99	KP256797.1	+	+	+	−
MF6	*Syncephalastrum racemosum* strain AQGSS 12	99	KP721597.1	+	−	−	−

Pathogenic fungi – F: *Fusarium oxysporum*; R: *Rhizoctonia solani*; S: *Sclerotinia sclerotiorum*; B: *Botrytis cinerea*.

Effect of endophytic fungi strains against plant pathogenic fungi: −Not active; + slightly active (< 50%).

#### Fermentation of isolated pure fungal strains

Large-scale cultivation of the isolated endophytic fungi was carried out in 1000 mL Erlenmeyer flask containing sterilized rice media as described by Kjer et al. (2009). Pure fungal strain (1–2 weeks growth on PDA and MEA) was cut into pieces and inoculated into one Erlenmeyer flask containing 200 g sterilized solid rice media. A flask of rice media without any inoculum served as a control. Cultivation was performed at room temperature under static condition for 3–6 weeks (depending on fungal growth) and the cultured flasks were examined periodically for possible contamination.

#### Extraction of cultured material

After the incubation period, the fermentation process was brought to an end with the addition of 250 mL of ethyl-acetate (EtOAc) to each culture flask. The culture media were cut into pieces with the aid of a glass rod and culture flask was placed on the shaker for 48 h to allow complete extraction. The mixture was filtered under vacuum using a Buchner funnel followed by repeated extraction with ethyl-acetate untill exhaustion. The EtOAc extract was concentrated using a rotary evaporator (Heidolph Inc, Germany) and the resulting extract was partitioned between *n*-hexane and 90% methanol (Kjer et al. 2009).

### Biological activity

#### Antagonistic activity of endophytic fungi strains

Dual culture technique was used to investigate the antagonistic effect of the endophytic fungi against four plant pathogenic fungi (Oldenburg et al. 1996). The assay was performed on potato dextrose agar as it favors the growth of the pathogenic fungi – *Fusarium oxysporum* (ITCC 3636), *Sclerotinia sclerotiorum* (ITCC 6323), *Botrytis cinerea* (ITCC 7478), and *Rhizoctonia solani* (ITCC 6882). Mycelia agar discs (5 mm diameter) from endophytic and pathogenic fungi cultures were inoculated on PDA plate at the periphery, opposite to each other. Petri plates inoculated only with test pathogens served as control. The experiment was done in triplicates. Paired cultures and control plates incubated at 24 °C for 5–7 days were observed and antagonism was expressed as percentage growth inhibition.

#### Cytotoxicity of fungal extracts against HeLa cancer cell line

The cytotoxic effect of the ethyl-acetate extract, hexane and methanol fractions of *M. tomentosa* fungal extracts against cervical (HeLa) cancer cell lines was screened using the MTT assay as earlier described by Ibrahim et al. (2013).

#### Antifungal activity of endophytic fungi extracts

The antifungal activity of the crude fungal extract along with the hexane and methanol fractions was tested against four plant pathogenic fungi, namely, *F. oxysporum* (ITCC 3636), *S. sclerotiorum* (ITCC 6323), *B. cinerea* (ITCC 7478), and *R. solani* (ITCC 6882) using the poisoned food technique (Kumar & Kaushik 2013). From a 40 mg/mL stock solution, 62.5 125 and 250 μL were taken by sterilized pipette and added to 10 mL molten potato dextrose agar in a test tube to obtain extract concentrations of 250, 500 and 1000 μg/mL. The solution was mixed thoroughly and then poured into sterilized Petri plate to solidify. With the aid of a cork borer, 5 mm mycelium block of each test fungus was inoculated at the centre of each Petri plate in an inverted position for greater contact of the mycelium with the culture medium. The plates were incubated at 25 ± 2 °C and diameter (mm) of fungal colony was measured at an interval of 24 h till the control plates attained the full growth. The media mixed with 250 μL of methanol served as the control growth plate. The experiment was done in three replicates. Percentage inhibition of mycelia growth of the test fungi by the extracts was calculated relative to the mycelia growth of the test fungi on the control plates.

### Antifeedant activity

#### Collection and rearing of test insect

Larvae of cotton armyworm, *Spodoptera litura,* were obtained from National Bureau of Agriculturally Important Insect (NBAII) with culture collection and identification code (NBAII-MP-NOC-02). In the laboratory, at room temperature (25 ± 2 °C) and 75% relative humidity, castor bean leaves (*Ricinus communis* L.) were provided for larvae feeding till pupal stage. After pupation, emerging adults were fed on 10% sucrose solution mixed with 1 mL of multivitamin syrup to enhance their reproductive ability. Healthy eggs produced by the adults were laid on folded filter paper placed in the cage. After the laying of eggs, egg masses were collected and allowed to hatch. The culture process was repeatedly maintained throughout the study period. The laboratory reared, dark green colour with yellow longitudinal lines on the dorsal surface, fifth in-star larvae were selectively preferred for the bioassay as they are voracious feeders.

#### Bioassay

The antifeedant activity of the crude extract and different solvent fractions of the fungal isolates was investigated by leaf disc choice test method (Akhtar & Isman 2004) with modifications. The test was performed in Petri plates (86 mm diameter) lined with 2% agar and divided into equal quadrants. A 9 mm diameter hole was drilled in each quadrant to provide support for the leaf disc. Leaf discs (9 mm diameter) were punched from fresh castor bean leaves and placed in each quadrant of the agar plate. With the aid of a micropipette, 10 μL of 1000 μg/mL of each of the fungal extracts in methanol (treated leaf disc) and methanol without the extract (control leaf disc) was spread alternately on the surface of the discs. The discs were air dried at room temperature for solvent evaporation. Two pre-starved (2 h) fifth-instar larvae of *S. litura* were gently placed at the centre of each Petri plate and covered. For each fungal extract, five replicates were maintained. The larvae were allowed to feed on both the treated and control leaf discs till approximately 75% consumption of either the treated or control (1–2 h) was achieved, after which the larvae were removed from the Petri plates. The unconsumed leaf discs were neatly pasted on a clear sheet of paper, scanned and the area was measured using ImageJ software (NIH, Bethesda, MD). The antifeedant activity was calculated using the formula:

$$\mathrm{Antifeedant}\;\mathrm{activity}=\frac{\mathrm{Leaf}\;\mathrm{consumed}\;\mathrm{in}\;\mathrm{the}\;\mathrm{control}\;\mathrm{disc}-\mathrm{Leaf}\;\mathrm{consumed}\;\mathrm{in}\;\mathrm{the}\;\mathrm{treated}\;\mathrm{disc}}{\mathrm{Leaf}\;\mathrm{consumed}\;\mathrm{in}\;\mathrm{the}\;\mathrm{control}\;\mathrm{disc}+\mathrm{Leaf}\;\mathrm{consumed}\;\mathrm{in}\;\mathrm{the}\;\mathrm{treated}\;\mathrm{disc}}\times 100$$

## Results and discussion

**Figure 1.**
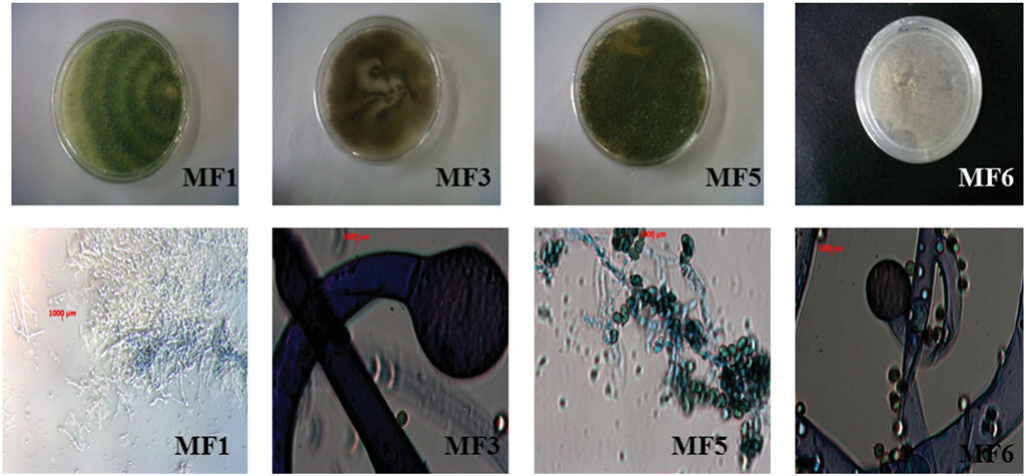
The morphology (colony appearance, fruiting structures and spores) of identified endophytic fungi isolated from the leaves of *Markhamia tomentosa*.

Six endophytic fungi were isolated from the leaf segments of *Markhamia tomentosa* inoculated on malt extract agar (MEA) and potato dextrose agar (PDA) media. To the best of our knowledge, this is the first report describing the isolation of endophytic fungi residing in *M. tomentosa* leaves. According to rDNA sequencing to their ITS region, four endophytic fungi isolates were successfully identified. Two endophytic fungal isolates, MF1 and MF5, were identified as *Trichoderma longibrachiatum* while isolates MF3 and MF6 were identified as *Syncephalastrum racemosum*. The blast percentage similarity to sequences in the NCBI database from previously identified fungi ranged from 98 to 99% (Table 1). Two fungal isolates MF2 and MF4 remained unknown due to the limitations inherent in DNA-based identification. The morphology of the identified isolates, including their fruiting structures and spores, is shown in Figure 1.

**Figure 2.**
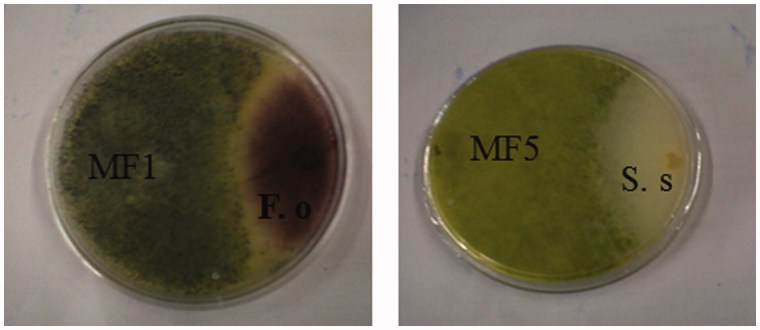
Dual culture bioassay of endophytic fungi of *Markhamia tomentosa* against *Fusarium oxysporum* (F. o; ITCC 3636) and *Sclerotinia sclerotorium* (S. s; ITCC 6323).

Many scientists have become increasingly interested in endophytic fungi as an alternative source in controlling plant and human pathogens. It is well-established that protection of the plant host against pests and pathogens is in the best interest of both endophytes and the host genome itself (Idris et al. 2013). In this study, a dual culture assay (Figure 2) was carried out to assess the antagonistic effects of the endophytic fungal strains isolated from *M. tomentosa* against four plant pathogenic fungi namely *F. oxysporum* (ITCC 3636), *S. sclerotiorum* (ITCC 6323), *B. cinerea* (ITCC 7478), and *R. Solani* (ITCC 6882). As shown in Table 1, *T. longibrachiatum* and *S. racemosum* isolates (MF1, MF3, MF5 and MF6) showed antagonistic activity against *F. oxysporum* (ITCC 3636). While *T. longibrachiatum* isolates (MF1 and MF5) showed antagonistic effect against *R. solani* (ITCC 6882) and *S. sclerotiorum* (ITCC 6323) (Table 1). None of the endophytic fungi isolates was effective against *B. cinerea* (ITCC 7478). Our results are however supported by a previous study which reported that endophytic *Trichoderma* spp isolated from the roots of C*offea arabica* antagonized *Fusarium* spp, and *S. sclerotiorum* (Mulaw et al. 2013).

All the fungal endophytes were cultivated on solid rice media at room temperature and extracted with ethyl acetate followed by partitioning between 90% methanol and *n*-hexane. The yield of the different solvent extracts obtained from each cultured material of the fungal isolates is shown in Table 2.

**Table 2.** Yield of extracts obtained from endophytic fungi isolated from *Markhamia tomentosa*.

		Yield of extracts (mg) in 200g of solid rice media		
Number	Isolate code	Ethyl acetate	Methanol	Hexane
1.	MF1	554	197	276
2.	MF3	180	93	21
3.	MF5	437	157	151
4.	MF6	480	298	55

Endophytes that produce host plant bioactive compounds with therapeutic value or potential have been reported in literature (Stierle et al. 1993; Amna et al. 2006; Shweta et al. 2010; Kusari et al. 2012) and it is possible that the endophytic fungi could be the original source of these bioactive products (Strobel & Daisy 2003). In our previous investigation (Ibrahim et al. 2013), *M. tomentosa* leaf extract inhibited cell viability and growth of HeLa cervical cancer cells with an IC_50_ value of 189.1 μg/mL. In this present study, the results of the *in vitro* cytotoxicity activity showed that crude fungal extracts of *T. longibrachiatum* isolates (MF1 and MF5) of *M. tomentosa* inhibited the proliferation of HeLa cancer cells with an IC_50_ value of 68 and 187.4 μg/mL (Table 3), respectively while that of *S. racemosum* isolate (MF3) gave an IC_50_ value of 137 μg/mL. *S. racemosum* isolate (MF6) gave an IC_50_ value greater than 250 μg/mL. Methanol fraction of *S. racemosum* isolate (MF3) on the other hand inhibited the cell-growth of HeLa cell line with an IC_50_ value of 43.56 μg/mL (Table 3). The IC_50_ obtained for the hexane extract of the isolate (MF3) was greater than 250 μg/mL. Our results suggest that the endophytic fungi residing in the leaves *M. tomentosa* are able to inhibit the cell viability and growth of HeLa cervical cancer cell lines.

**Table 3.** Antiproliferative, antifungal and antifeedant activity of fungal crude extracts and fractions.

	Inhibition of cell growth	Poisoned food assay @ 1000 μg/ml				%Antifeedant activity
Fungal isolates	HeLa cell line (IC_50_)	F	R	S	B	*Spodoptera litura*
MF1: ethyl acetate	68 μg/ml	+	+	+	−	−
Methanol	>1000 μg/ml	+	+	+	−	−
Hexane	>2000 μg/ml	+	−	−	−	+
MF3: ethyl acetate	137 μg/ml	+	−	−	−	−
Methanol	43.56 μg/ml	+	+	+	−	−
Hexane	>250 μg/ml	−	−	−	−	−
MF5: ethyl acetate	187.4 μg/ml	+	+	+	−	−
Methanol	>250 μg/ml	+	+	+	−	−
Hexane	>1000 μg/ml	−	−	−	−	−
MF6: ethyl acetate	>250 μg/ml	−	−	−	−	+
Methanol	ND	+	−	−	−	−
Hexane	ND	−	−	−	−	−

Pathogenic fungi – F: *Fusarium oxysporum*; R: *Rhizoctonia solani*; S: *Sclerotinia sclerotiorum*; B: *Botrytis cinerea*.

Effect of fungal crude extracts and fractions against plant pathogenic fungi and *S. litura*; (−): Not active; (+): slightly active (<50%).

In the poisoned food assay, the ethyl acetate extracts and methanol fractions of *T. longibrachiatum* isolates (MF1 and MF5) and the methanol fraction of *S. racemosum* isolates (MF3) showed a MIC value of 1000 μg/mL (Table 3). As observed with the dual culture assay, none of the extracts and fractions of the fungal isolates showed antifungal activity against *B. cinerea* (ITCC 7478). The results obtained from this assay correlate with previous study which reported the antimicrobial activity of extract of *Syncephalastrum* spp. isolated from *Adathoda beddomei* against fungi and bacteria (Prabavathy & Valli 2013).

Although endophytic fungi are known to deter insect pests (Clay 1989; Carroll 1991, 1995; Azevedo et al. 2000; Gond et al. 2010), only minimal feeding deterrent activity (<50%) against *S. litura* was recorded in hexane fraction of *T. longibrachiatum* MF1 isolate and *S. racemosum* ethyl acetate extract of MF6 isolate (Table 3).

## Conclusion

This study demonstrated that the leaves of *M. tomentosa* harbor strains of endophytic fungi of promising plants and human health benefits. The results indicated that *Trichoderma longibrachiatum* and *Syncephalastrum racemosum* (fungal isolates of *M. tomentosa*) may be useful in protecting plants from pathogenic fungi. It may be learned that the pharmacological effect of a medicinal plant, may not be due to the natural products of the plant, but of the endophytes inhabiting the plant (Strobel & Daisy 2003). The fungal isolates in this study, exhibited interesting antiproliferative effect on HeLa cells as observed by our previous work on the plant. Therefore, further research on both the endophytic fungi and host plant, to isolate and identify the bioactive compounds responsible for the claimed biological activity is on-going.
